# Obesity and cancer: Methodological frontiers for mechanistic discoveries

**DOI:** 10.1371/journal.pmed.1005081

**Published:** 2026-04-24

**Authors:** Steven C. Moore, Patrick J. Ryan

**Affiliations:** Division of Cancer Epidemiology and Genetics, National Cancer Institute, National Institutes of Health, Rockville, Maryland, United States of America

## Abstract

Why obesity increases cancer risk has long remained a biological mystery. In this Perspective, Steven Moore and Patrick Ryan discuss rapid advances in statistical and analytical methods that are now opening the door to mechanistic discoveries that may finally help resolve this question.

## Teaser

It has long been a biological mystery why obesity increases cancer risk. Rapid advances in statistical and analytical methods are now opening the door to mechanistic discoveries that may finally resolve this question.

Obesity is an established risk factor for at least 13 types of cancer [1], yet *how* it causes cancer remains a central unresolved question. The challenge is as much methodological as biological; adipose tissue is a dynamic endocrine organ that perturbs thousands of interrelated biological factors simultaneously [2]. Traditional reductionist approaches—isolating one factor at a time, often in animal models—are poorly matched to this complexity, and experimental systems can fail to capture key features of human cancer biology. Progress, therefore, depends on developing efficient ways to screen candidate mediators in their native human context. Epidemiologic studies that pair high-throughput molecular profiling with mediation analyses offer perhaps the clearest path toward that goal.

## A methodological and technological shift

**Fig 1:**
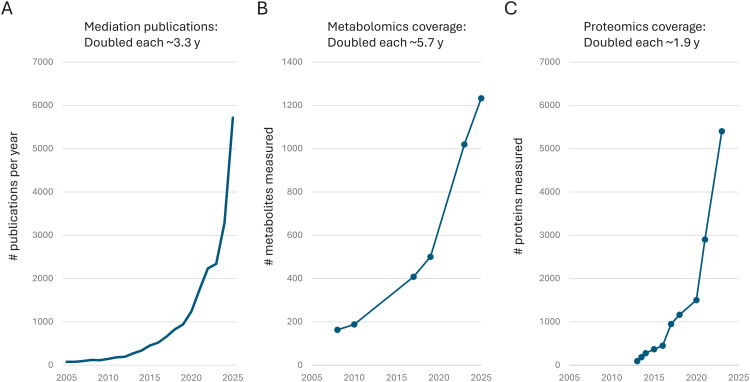
Methodological and technological advances shaping mechanistic research, 2005–2025. **A.** Growth in mediation-related publications in PubMed, 2005–2025. Title/abstract search terms of “mediation analysis”, “causal mediation”, or “mediated effect”. Key milestones include VanderWeele 2009 [3], which helped standardize and popularize the indirect/direct effects framework, and VanderWeele 2015 [4], which made the framework more teachable and accessible through software. From 2017 onward, growth accelerated, possibly reflecting increased use of mendelian randomization and the incorporation of high-throughput data into UK Biobank and other prospective cohorts. **B.** Growth in the number of metabolites measured by metabolomics platforms, as exemplified by Biocrates’ primary platform, 2005–2025 (see [5] for underlying data). Other metabolomics platforms experienced similar growth, e.g., Metabolon’s coverage increased from low hundreds of metabolites in 2010 to low thousands by 2020. **C.** Growth in the number of proteins measured by proteomics platforms, as exemplified by Olink’s platforms, 2005–2025 (see [5] for underlying data). Other proteomics platforms experienced similar growth, e.g., Somalogic’s coverage increased from 1,300 in 2015 to 11,000 in 2024.

Mediation analysis is a set of methods used to assess whether, and to what extent, an exposure affects an outcome through an intermediate variable or pathway. Over the last two decades, advances in mediation methodology and high-throughput molecular assays have opened new frontiers for mediation research in epidemiology. As mediation frameworks were formalized and implemented in accessible software [3,4], their application in biomedical research surged (Fig 1A). In parallel, “omics” platforms—such as metabolomics (Fig 1B) and proteomics (Fig 1C)—dramatically expanded their analytical coverage, enabling increasingly high-dimensional screens of candidate mediators. Meanwhile, mendelian randomization (MR), an analytical approach that uses genetic variants as instrumental variables to minimize confounding and reverse causation, has made it possible to interrogate candidate mediators with genetic proxies in large genomics consortia. These collective advances mark a consequential, and still underappreciated, shift in modern epidemiology.

Two recent studies in *PLOS Medicine* by Alcala and colleagues [6] and Li and colleagues [7] illustrate this shift. Focusing on kidney and liver cancer, respectively, these studies offer a chance to evaluate where mediation analyses stand amid rapid technological advances, and to consider where they might head next.

Of these studies, Alcala and colleagues adopted the more traditional, hypothesis-driven strategy [6]. Guided by long-standing hypotheses [8], they evaluated 17 hormonal, metabolic, inflammatory, and cardiometabolic biomarkers as candidate mediators of the adiposity-kidney cancer association in two prospective cohorts (UK Biobank and the Northern Sweden Health and Disease Study) and complemented these analyses with MR of the same biomarkers. Across observational and MR analyses, five biomarkers—insulin, diastolic blood pressure, triglycerides, high-density lipoprotein cholesterol, and sex hormone–binding globulin—emerged as partial mediators. Yet the estimated mediation was modest (e.g., 22% for insulin), comparable to prior findings for other cancers [9]. The study advanced the field by testing canonical hypotheses at scale and greatly strengthened claims for causality through MR. However, it did not fully resolve the mediating factors, perhaps because targeted approaches are liable to miss important mediators.

In contrast, Li and colleagues pursued a more discovery-driven strategy, using metabolomics in the Shanghai Men’s Health Study to screen 186 circulating metabolites as candidate mediators of the adiposity–liver cancer association [7]. Twenty-seven metabolites, including bile acids, organic acids, and multiple amino acids, were associated with at least one adiposity measure and with liver cancer risk, meeting the authors’ criteria for candidate mediators. Several of the liver cancer associations were strikingly large, with odds ratios of three or more per standard deviation increase in the log-transformed concentrations of certain bile acids. Notably, the study reported near-complete mediation: after accounting for key metabolite mediators, adiposity showed no residual direct association with liver cancer risk. Some findings have been reported previously (e.g., bile acids and tyrosine) [10], but the authors extend these observations to a non-European population, identify novel candidates, and organize the implicated metabolites into coherent biological pathways. In doing so, the study offers a vivid demonstration of the value of high-dimensional mediation screens, though its discovery-oriented design means the findings still require replication and follow-up.

Emerging data suggest that proteomics may offer even greater discovery potential. Adiposity correlates with thousands of circulating proteins, sometimes profoundly so, as in the strong correlation between Body Mass Index and leptin [2]. More importantly, recent studies suggest that proteomic profiles are powerfully associated with future cancer risk [11,12]. Among the implicated proteins is Hepatitis A Virus Cellular Receptor 1 (HAVCR1), one of the strongest kidney cancer biomarkers yet identified. HAVCR1—and the biological pathways it represents—is now thought to substantially mediate the link between obesity and kidney cancer risk; its biology specifically implicates chronic proximal tubule injury as a key underlying mechanism [13]. Comparable signals have also been reported for liver cancer, multiple myeloma, non-Hodgkin lymphoma, and several other obesity-associated cancers. This abundance of candidate mediators suggests that mechanisms once viewed as biologically intractable are now becoming analytically tractable through high-throughput omics.

## Mediation in the GLP-1 era

As mediation methods matured, obesity treatment itself underwent a revolution. The rise of incretin-based pharmacotherapies, including glucagon-like peptide 1 (GLP-1) receptor agonists and emerging dual- and triple-agonist agents, has led to weight loss so substantial that it has prompted some pundits to speculate that we may be able to “treat our way out” of the obesity epidemic [14].

But does such therapeutic success render mechanistic understanding unnecessary? On the contrary—it heightens the urgency.

Presently, the medical system is grappling with how broadly to prescribe incretin-based therapies, weighing their potential health benefits against costs and side effects. Reduced cancer risk may be among the benefits, but without mechanistic clarity, neither the magnitude nor the timing of any such effect can be confidently projected. For a public health question with such high stakes, mechanistic precision is needed to ground causal claims and make them persuasive.

In epidemiology, this is an old lesson. In 1847, Ignaz Semmelweis showed that handwashing reduced childbirth mortality at Vienna General Hospital by 90%, yet his findings were largely dismissed because they lacked an accepted mechanism; germ theory had not yet arrived. Mechanistic precision matters for a second reason: without it, interventions can backfire. . In 1840s London, health reformers correctly recognized human waste as central to cholera, but they misidentified the mode of transmission, attributing it to noxious odors under the miasma theory. Their solution—flushing cesspits into the Thames—channeled pathogens into the public water supply, accelerating the spread of cholera and converting a nuisance in the nose into a hazard on the lips.

In brief, a precise understanding of mediation is vital to turning observations and associations into meaningful public health actions. In exploring the mechanisms linking obesity and cancer, these studies [6,7] point the way forward, demonstrating how statistical and analytical advances enable mechanistic discoveries that could finally help resolve the long-standing mystery of obesity’s association with cancer risk.

## Abbreviations

GLP-1: glucagon-like peptide 1
HAVCR1: Hepatitis A Virus Cellular Receptor 1
MR: mendelian randomization.
